# Coupling clinical exome sequencing with functional characterization studies to diagnose a patient with familial Mediterranean fever and *MED13L* haploinsufficiency syndromes

**DOI:** 10.1002/ccr3.904

**Published:** 2017-04-18

**Authors:** Sureni V. Mullegama, Phillip Jensik, Chen Li, Naghmeh Dorrani, UCLA Clinical Genomics Center, Sibel Kantarci, Bruce Blumberg, Wayne W. Grody, Samuel P. Strom

**Affiliations:** ^1^ UCLADepartment of Pathology and Laboratory MedicineDavid Geffen School of MedicineUniversity of California, Los AngelesLos AngelesCalifornia; ^2^ UCLA Clinical Genomics CenterDavid Geffen School of MedicineUniversity of California, Los AngelesLos AngelesCalifornia; ^3^ Department of PhysiologySouthern Illinois University School of MedicineCarbondaleIllinois; ^4^ Department of Cellular and Genetic MedicineSchool of Basic Medical SciencesFudan UniversityShanghaiChina; ^5^ Department of Human GeneticsDavid Geffen School of MedicineUniversity of California, Los AngelesLos AngelesCalifornia; ^6^ Department of PediatricsDavid Geffen School of MedicineUniversity of California, Los AngelesLos AngelesCalifornia; ^7^ Kaiser PermanenteOaklandCalifornia

**Keywords:** Clinical exome sequencing, *DEAF1*, familial Mediterranean fever, *MED13L*, *MED13L* haploinsufficiency syndrome, *MEFV*

## Abstract

Clinicians should consider that clinical exome sequencing provides the unique potential to disentangle complex phenotypes into multiple genetic etiologies. Further, functional studies on variants of uncertain significance are necessary to arrive at an accurate diagnosis for the patient.

## Introduction

The introduction of next‐generation sequencing (NGS), particularly clinical exome sequencing (CES), has exponentially expanded the identification of known and novel genes in patients with suspected genetic disorders 1, 2. CES is a powerful tool to interrogate the exome and enables examination of coding regions of the genome for variants in genes associated with Mendelian disorders and those yet to be linked to a genetic disorder. CES has a diagnostic advantage over other genetic tests because of several reasons: (1) Single gene or NGS panels might not be developed for gene of interest (1) genetic heterogeneity, (2) incomplete or atypical clinical presentation, and (3) lack of knowledge of the causal gene. One perhaps underappreciated power of this gene‐agnostic approach is its ability to identify multiple root causes of a complex set of clinical symptoms in a single patient 2. However, this can lead to uncertainty in definitively determining the individual contribution of each variant, if any, to disease pathogenesis and phenotype. One logical but underused solution to this problem is to couple CES with thorough phenotypic assessments and functional characterization studies of the variants.

Herein, we illustrate how a complex phenotypic case that presents with daily fevers, intellectual disability (ID), autism spectrum disorder (ASD), and behavioral problems can be potentially solved through clinical exome sequencing, comprehensive phenotypic assessments, and functional characterization of these variants.

## Case Report

At the time of the last evaluation, the patient was an 8‐year‐old boy who was born following an uneventful pregnancy to nonconsanguineous parents of European and Filipino descent, who were clinically normal. He was born at full‐term gestation weighing 3.49 kg. At birth, he was noted to have right‐sided torticollis with an unusual shape to the right ear. His infancy was marked by suspected atonic or absence seizures, periodic fevers, sleep disturbance, feeding difficulties, daily episodes of choking and wheezing, frequent respiratory infections, and abnormal development. At 6 months of age, he had 5 weeks of daily diarrhea episodes, which led to a metabolic workup including the following biochemical tests: urine organic acid screen, plasma amino acid screen, acylcarnitine, uric acid, urine vanillylmandelic acid, urine dopamine, and 24‐h urine metanephrines, all of which were all normal. He was enrolled in a sleep study due to his sleep disturbances which revealed a diagnosis of obstructive and central sleep apnea. The patient walked at 14 months. At 15 months, behaviors such as head banging, crying, unprompted violent tantrums, and banging toys to his head occurred. He was evaluated by ophthalmology at 15 months for unilateral right eye strabismus and was diagnosed with bilateral temporal optic nerve atrophy. At 2 years of age, he was evaluated by a psychologist who felt that the patient met the criteria for autism; he was officially diagnosed with ASD at 35 months. He began to receive regular physical therapy, occupational therapy, speech therapy, and applied behavioral analysis. At 3 years of age, the patient still continued to have daily fevers which would range from 99 to 102 F°, and peritonitis‐like abdominal pain. Further, he had a high threshold of pain, and poor social interaction skills. At 5 years of age, a physical examination noted asymmetric facies with simple uncurled slightly low‐set right ear that protrudes from the head, enlargement or protrusion of the skull, and two small *café au lait* spots. He continued to have anxiety and disruptive and aggressive behavioral issues which were treated with guanfacine and citalopram. At 8 years of age, his speech was more fluent, repetitive behaviors were prevalent, decreased eye contact was noticed, body was somewhat rigid at times, and he had normal attention and concentration.

## Methods

### Patient ascertainment

The affected boy and his adopted parents were seen at the Kaiser Permanente Oakland Genetics Clinic. Clinical assessment included a review of medical records, including developmental, biochemical, neurological, and genetic evaluations. Fresh peripheral blood samples for gene expression analysis were collected from the proband, his adopted mother, and clinically normal age‐ and sex‐matched controls (*n* = 3). Informed consent was obtained from all participants. This study was approved by our Institutional Review Board at University of California, Los Angeles.

### Clinical exome sequencing

Clinical exome sequencing was performed using standard protocols from genomic DNA extraction to data analysis 27 1 in our CLIA‐certified laboratory. Briefly, DNA was extracted from proband using standard methods. Exome capture was performed using Agilent SureSelect XT Clinical Research Exome, sequencing was performed on HiSeq2500 as 100‐bp paired‐end run, and variant annotation was performed using GoldenHelix SNP & Variation Suite. In total, 8,191,699,021 bases of sequence were generated and uniquely aligned to both the human reference genome and mitochondrial genome, generating a mean coverage of 92.53x per base within the RefSeq protein coding bases of the human genome. Overall, the data were consistent with a 46, XY, male. The data were consistent with a high‐quality genomic sequence and fall within normal human genomic variation quality parameters. We estimate from these data that about 98.4% of the known disease‐causing bases were reliably sequenced with at least 10x coverage. If the candidate variant had a quality score of ≥Q500, confirmatory Sanger Sequencing was not deemed necessary 3.

### CES data analyses

Basic demographic information, ordering physician specialty, family history, and phenotype information were obtained from submitted clinical information. The phenotype that was in the reporting clinicians' note was collected at enrollment and recorded using PhenoTips. A phenotype‐driven list of candidate genes was labeled as our primary gene list. This list was created by matching the phenotypes against Online Mendelian Inheritance in Man (OMIM) and Human Gene Mutation Database (HGMD). The variants in the primary gene list were prioritized first for our data analysis. Variants outside the primary gene list were also analyzed if nothing was identified in the primary gene list. Variant classification was consistent with guidelines set forth by the American College of Medical Genetics and Genomics (ACMG) 4. Variant classifications were reviewed in our genomic data board, which is a multidisciplinary team consisting of clinical geneticists, other medical subspecialists, genetic counselors, molecular geneticists, and bioinformaticians.

### Functional studies

#### Plasmids

DEAF1‐FLAG expression constructs have been previously described 5. A gBlock^®^ (Integrated DNA Technologies) containing the c.688C>G variant and specific DEAF1 native restriction endonuclease sites was used to replace the corresponding region of wild‐type (WT) DEAF1 to introduce the p.Q230E amino acid substitution. The reporter plasmid pDEAF1pro‐luciferase and DEAF1 p.Q264P expression construct have been previously described 6. All plasmid constructs were confirmed by DNA sequencing by a Beckman Coulter CEQ 8000 Genetic Analysis System.

### Electrophoretic mobility shift assay

Wild‐type, p.Q230E, and p.Q264P DEAF1 carboxy‐terminal FLAG‐tagged proteins were purified from transfected human embryonic kidney 293T (HEK293T) cells as previously described 7. WT or mutant DEAF1 proteins (850 fmol) were incubated with 500 fmol of infrared dye‐labeled N52‐69 (IR800) or S6con (IR700) dsDNA probe 6 with 1 *μ*g of poly(dA‐dT) in a 20‐*μ*L reaction containing 70 mmol/L KCl, 35 mmol/L Tris, pH 7.5, 0.7 mmol/L DTT, and 2% (v/v) glycerol, for 20 min at 25°C. Protein–DNA complexes were separated by electrophoresis on 5.0% nondenaturing polyacrylamide gels and bands were detected by a Li‐Cor Odyssey CLx infrared scanner.

### Subcellular localization

HEK293T cells on 35‐mm dishes were transfected with 500 ng of p.Q230E FLAG‐tagged DEAF1. Twenty‐four hours later, cells were fixed in paraformaldehyde, permeabilized in methanol, and incubated with rabbit anti‐DEAF1 28 (1:1,000) followed by Cy3‐conjugated goat anti‐rabbit IgG. DNA was stained with Hoechst dye 33258 (1 *μ*g/mL). Cells were visualized with an Olympus BW50 fluorescence microscope with a 60x water objective.

### Transcription assay

Transcription assays were performed as previously described 6.

### Gene expression analysis

Total RNA was isolated according to standard methods (Invitrogen, Carlsbad, CA), quantified using the NanoDrop^®^ ND‐100 Spectrophotometer, and reverse‐transcribed with qSCRIPT cDNA SuperMix (Quanta Biosciences, Inc., Gaithersburg, MD) according to the manufacturer's instructions. Real‐time quantitative PCR (RT‐qPCR) with Taqman probes (Life Technologies, Carlsbad, CA) for *MED13L* (Hs00392609_m1), and *GAPDH* (Hs9999905_m1) was performed as previously described 8. Multiple comparisons were performed using a one‐way or two‐way ANOVA and Tukey's post hoc correction where appropriate. Statistical significance was determined at *P* < 0.05.

## Results

### Genetic testing

Due to the wide variety of symptoms, a single unifying diagnosis was not apparent. Thus, our patient went through extensive genetic testing. Standard chromosome analysis (karyotype, and chromosomal microarray analysis) yielded normal results. Other genetic testing, fragile X syndrome testing, and metabolic testing were normal as well. Due to the periodic fevers, a NGS periodic fever syndrome panel was performed which consisted of seven genes (*ELA2*,* LPIN2*,* MEFV*,* MVK*,* NLRP3*,* PSTPIP1*, and *TNRSF1A*) (GeneDx, Gaithersburg, MD). The patient was found to have three well‐known and classified heterozygous variants in the familial Mediterranean fever gene (*MEFV;* MIM 608107) (Table S1). The first two pathogenic *MEFV* mutations p.Pro369Ser and p.Arg408Gln are in strong linkage disequilibrium, and typically found in *cis* on a single chromosome 9, 10, 11. The third *MEFV* variant, p.Glu148Gln, is a disease‐associated polymorphism and is a mild mutation commonly associated with atypical presentations rather than with classical FMF symptoms 12. While the geneticist felt that the patient's symptoms were atypical for classic FMF, a therapeutic trial of colchicine was conducted which did alleviate his fever and pain attacks, thus confirming the diagnosis on a functional basis 13, 14.

**Figure 1:**
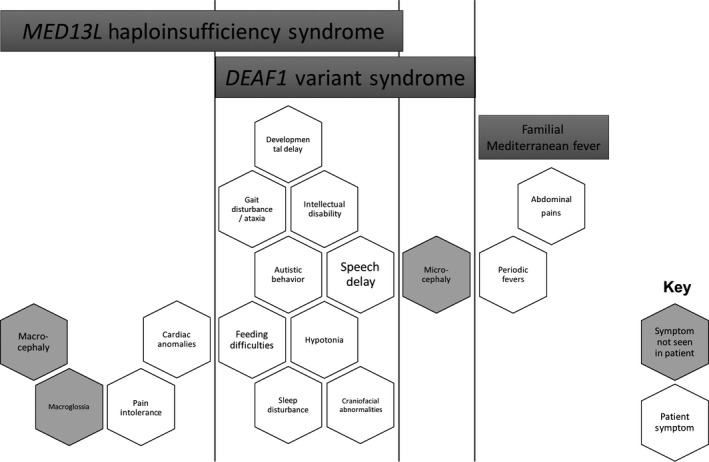
Phenotype Comparison of Patient with *MED13L* haploinsufficiency syndrome, *DEAF1‐*associated syndrome, and familial Mediterranean fever syndrome. Each hexagon represents a symptom associated with one or more of the diseases, as indicated by the bars at the top of the figure. Those symptoms observed in the affected participant are open (filled white) and those not observed are filled gray.

As a diagnosis of FMF could not explain the entire phenotype of our patient, specifically the neurological features, CES was performed. In total, 23,654 DNA variants were identified within the coding exons ± 2 bp in the proband with no regions of homozygosity (>5 Mb) observed. A primary gene list from the Human Gene Mutation Database (HGMD) and Online Mendelian Inheritance in Man (OMIM) was generated based on the clinical presentation and phenotype to prioritize the variants. Within the primary gene list, we confirmed the previously identified pathogenic *MEFV* variants from the NGS fever panel. In total, six homozygous and 414 rare heterozygous protein‐altering variants of uncertain clinical significance (VUS) were identified across 401 genes, which included two heterozygous variants of interest. We applied the guideline for interpretation of sequence variants recommended by the American College of Medical Genetics and Genomics (ACMG) to classify these two heterozygous variants 4 (see Table S1). The first variant was in the Deformed Epidermal Autoregulatory Factor‐1 (*DEAF1*; MIM 602635), gene which encodes a transcriptional factor with an important role in central nervous system and early embryonic development 29. Variants in the *DEAF1* gene are associated with autosomal dominant intellectual disability‐24 (MRD24, MIM 615828) 6. The *DEAF1* variant, c.688C>G (p.Gln230Glu), leads to a missense change affecting a well‐studied functional domain called the SAND domain (Fig. 1A and Table S1). De novo mutations affecting the SAND domain of *DEAF1* are shown to cause intellectual disability with severe speech impairment and behavioral problems 6, 15, 16. The variant was not present in the Exome Aggregation Consortium (ExAC) reference data set (http://exac.broadinstitute.org) or 1000 Genomes Project (www.1000genomes.org) (Table S1). While the amino acid position is evolutionarily conserved, the conclusions of in silico prediction tools (SIFT and PolyPhen) were conflicting (Table S1). Based on this information and using the ACMG variant guidelines, we determined that the variant is likely pathogenic (Table S1).

The second variant, in the Mediator Complex Subunit 13‐like (*MED13L*; MIM 608771 gene, c.5282C>T (p.Prol1761Leu), is also a rare variant that has not been reported before and is absent from the ExAC and 1000 Genomes databases. Loss‐of‐function changes in *MED13L* are responsible for *MED13L* haploinsufficiency syndrome (MIM 608771) 17, 18. The variant is located in a conserved C‐terminal domain of *MED13L* 18. The in silico analysis by two algorithms (SIFT and PolyPhen2) was predicted to be tolerated/benign (Table S1). Overall, using the ACMG classification guidelines, *MED13L* was categorized as a VUS. Solely using the *ACMG* variant classification guidelines, the *DEAF1* variant had a higher pathogenicity score compared with the *MED13L* variant (Table S1). However, the UCLA Genomic Data Board was not convinced that the *DEAF1* variant was the sole contributor to our patient's neurological and behavioral phenotype.

### Phenotype assessment and comparison

Comparing the phenotype of our patient to the core phenotypes of *MED13L* haploinsufficiency syndrome and *DEAF1*‐associated syndrome, it was apparent that both of these syndromes could fit with our patient's phenotype 6, 19 (Fig. 1). However, based on the most recent summary of *DEAF1* patients, these patients have a more severe motor delay, severe delay in expressive speech (absent speech to ten single words), and very severe intellectual disability compared with our patient 6. Closer examination of the *MED13L* haploinsufficiency syndrome phenotype, through analyzing the 21 known reported *MED13L* variant cases 17, 19, 20, 21, 22, 23, 24, 25, 26, showed that our patient is similar to other *MED13L* haploinsufficiency cases (Table S2). Further, similar missense mutations of *MED13L* that occur later in the protein in the C‐terminal region have a milder phenotype in terms of ID and language development 18, 22. Finally, *MED13L* and *DEAF1* are both strong candidate autism genes according to the SFARI autism database (https://sfari.org/). Thus, while *DEAF1* had a higher variant pathogenicity score than *MED13L*, the patient's phenotype aligned more closely with *MED13L* haploinsufficiency syndrome (Fig. 1).

### Functional studies

**Figure 2:**
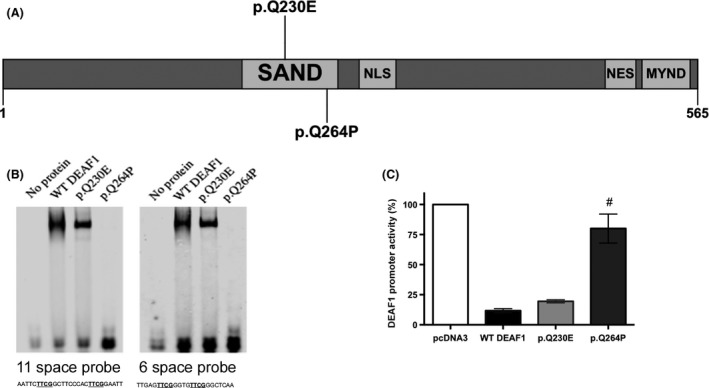
*in vitro* functional studies of the *DEAF1* variant (p.Q230E) (A) A schematic of DEAF1 (565 domains) and the locations of a known pathogenic *DEAF1* variant (p.Q264P) in gray and our proband's *DEAF1* variant (p.Q230E) in yellow. (B) EMSA of WT and altered FLAG‐tagged DEAF1 recombinant proteins isolated from HEK293T cells. Fluorescently labeled DNA ligands with a spacing of 11 or 6 nucleotides between the CG dinucleotides were examined. The positive control protein, p.Q264P proteins, lacked DNA binding, whereas the p.Q230E protein showed similar binding to the WT DEAF1. (C) *DEAF1* promoter activity after transfection with either WT or altered FLAG‐tagged DEAF1. The p.Q230E substitution had no effect on transcriptional repression. The p.Q264P served as a positive control of a known deleterious DEAF1 variant. Each bar represents the mean ± SEM of the normalized luciferase activity of three independent experiments when the activity of pcDNA3 (*DEAF1* promoter alone) was set to 100%. One‐way ANOVA with both Dunnett's multiple comparisons and selected Bonferroni post‐test of WT DEAF1 versus each mutant, ^#^ *P* < 0.001.

Since utilizing ACMG variant guidelines and conducting a phenotypic assessment and comparison evaluation of our patient did not firmly establish which variant was most likely pathogenic, we conducted a series of simple in vivo functional studies on the *DEAF1* and *MED13L* variants. To determine whether the proband's DEAF1 p.Q230E variant, which affects the SAND domain, could be the underlying cause of the neurological phenotype, we assessed the physiological consequences of this variant by using previously established functional studies 6. A known pathogenic DEAF1 variant (p.Q264P) 6 served as our negative control (Fig. 2A). In transfected cells, the p.Q230E variant showed overexpression and nuclear localization similar to the localization previously observed for WT DEAF1 (Fig. S1). As the SAND domain mediates *DEAF1* DNA binding, we performed an EMSA assay to examine whether impaired DNA binding could impair the transcriptional binding (Fig. 2B). The p.Q230E variant had similar DNA binding to the wild‐type DEAF1, whereas p.Q264P variant showed complete loss of DNA binding to both DNA probes (Fig. 2B). It has been shown that *DEAF1* represses its own *DEAF1* promoter, and mutations affecting the SAND domain can eliminate DNA binding and promoter repression 6, 7. In the reporter assay, the p.Q230E variant was functionally similar to WT; however, p.Q264P lost the ability to repress the *DEAF1* promoter (Fig. 2C).

**Figure 3:**
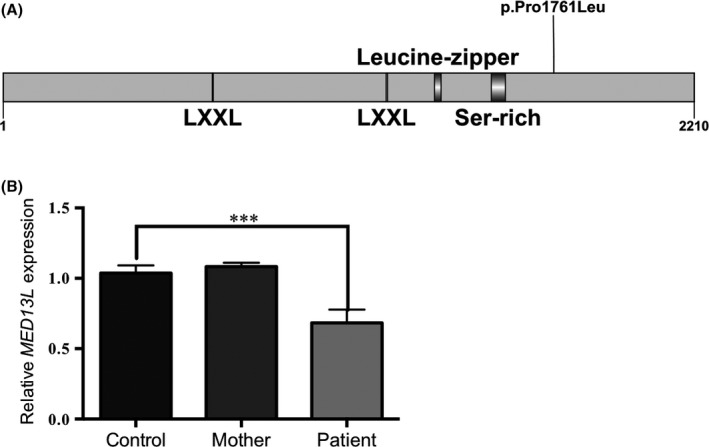
*MED13L* mRNA expression in whole blood from proband and control individuals. (A) Domain structure of MED13L. (B) Quantitative RT‐PCR was used to test *MED13L* mRNA expression levels. *MED13L* expression levels are not significantly altered in the mother compared with controls. *MED13L* mRNA expression levels are significantly decreased in our patient compared with controls. All results were normalized to *GAPDH* expression. Relative expression values were based on the ΔΔCt value. Expression of all controls was normalized to 1. Each bar represents means (±SEM) of values from 3 to 6 independent experiments. Multiple comparisons were performed using an ANOVA and Tukey's post hoc correction. Statistical significance was determined at *P* < 0.05. Asterisk indicates significance, ***P < 0.0001.

We next accessed the effect of the *MED13L* variant on mRNA expression of *MED13L* in our patient. We found that *MED13L* mRNA expression levels were significantly reduced compared with controls and the normal adoptive parent (*P* = 0.0005) (Fig. 3B). The patient's *MED13L* mRNA expression levels are consistent with levels expected for a patient with *MED13L* haploinsufficiency syndrome (Fig. 3B) 26.

## Discussion and Conclusions

We present an 8‐year‐old boy with a complex phenotype found to carry five missense variants in three different genes: *MEFV* (p.Pro369Ser, p.Arg408Gln, p.Glu148Gln), *DEAF1* (p.Gln230Glu), and *MED13L* (p.Prol1761Leu), identified through NGS and CES. The *MEFV* variants were known pathogenic variants and explained the daily fevers and peritonitis‐like abdominal pain that the boy had. In regard to the two other variants, we used the ACMG variant assessment guidelines. The *DEAF1* variant appeared a more attractive candidate than the *MED13L* variant. However, the clinical conditions associated with the disruption of *DEAF1* and *MED13L* genes shared overlapping core phenotypes due to these two genes being highly associated with autism. Thus, it was uncertain whether both variants could be contributing to the phenotype of our patient. Consequently, we conducted phenotype comparison studies and functional characterization of the *DEAF1* and *MED13L* variants to establish a more certain clinical diagnosis.

Initially, the *DEAF1* variant appeared to be the more obvious candidate than the *MED13L* variant when applying the ACMG variant assessment guidelines. By completing in vitro functional characterization of the two variants and some further phenotyping studies, we were able to show that our patient likely has a diagnosis of *MED13L* haploinsufficiency syndrome, in addition to familial Mediterranean fever (Figs. 2 and 3). We do have to be cautious in ruling out the *DEAF1* mutation because while the mutation does not affect DEAF1 function in the functional assays we used here (Fig. 1B–D), the variant may affect other facets of DEAF1 activity not assessed by our tests. Further, there may be other DEAF1 functions that have yet to be determined. The child is currently on colchicine which alleviates his fever and pain attacks that are symptoms of familial Mediterranean fever syndrome. To alleviate the behavioral issues that are due *MED13L* haploinsufficiency syndrome, he is treated with guanfacine and citalopram. Finally, he continues to receive speech‐language therapy, occupational therapy, and physical therapy which are all common in children with autism spectrum disorders.

Our study provides a cautionary tale in showing that we cannot always rely solely on the ACMG variant calling guidelines in determining pathogenicity in cases with complex etiologies such as ASD. Additionally, some of the key ACMG criteria on determining pathogenicity of a variant, such as localization in a known functional domain, various in silico predictions, and population frequency data, can be unreliable, especially in complex phenotypic cases. In retrospect, CES does force us to diagnostically think in a less linear manner and opens the possibility of explaining a patient's aggregate phenotype through multiple genetic disorders. Overall, this case highlights the unique potential of CES to disentangle complex phenotypes into multiple genetic etiologies and the need for functional characterization of variants to determine pathogenicity in complex and interacting disorders.

## Authorship

SVM: conceived the study, conducted the experiments, analyzed the data, created the figures, and wrote the manuscript. PJ: conducted the DEAF1 experiments, analyzed the data, and helped write the manuscript. CL: helped conduct the experiments. ND: helped with obtaining patient clinical information. UCLA Clinical Genomics Center: conducted the CES of the specimens. BB: saw the patient and wrote the clinical note. SK: analyzed/reported on the CES case. SPS: created a figure and helped write the manuscript. SVM, PJ, CL, BB, SK, WG, and SS: all edited the manuscript.

## Conflict of Interest

None declared.

## Supporting information


**Table S1.** *MEFV*,* DEAF1* and *MED13L* variants were identified through clinical exome sequencing.
**Table S2**. Features of patient compared to *MED13L* cases in the literature.
**Figure S1**. Subcellular localization studies.
