# Supplementary material for: Coupling clinical exome sequencing with functional characterization studies to diagnose a patient with familial Mediterranean fever and MED13L haploinsufficiency syndromes
Source: Clin Case Rep. 2017 Apr 18;5(6):833–40. doi: 10.1002/ccr3.904 (PMC5458005; doi:10.1002/ccr3.904)
Supplement: Supplementary file 1 — Table S1. MEFV, DEAF1 and MED13L variants were identified through clinical exome sequencing. Table S2. Features of patient compared to MED13L cases in the literature. Figure S1. Subcellular localization studies. [file CCR3-5-833-s001.docx]

Clinical Exome Sequencing with Functional Studies Diagnoses a Patient with Familial Mediterranean Fever and *MED13L* Haploinsufficiency Syndromes

S. V. Mullegama^1,2^*,* P. Jensik^3^, Li Chen^4^, N. Dorrani^1,2,5,6^ , UCLA Clinical Genomics Center^2^, S. Kantarci^1,2^ , B. Blumberg^7^, W.W.Grody^1,2,5,6^, S.P.Strom^1,2^

^1^UCLA, Department of Pathology and Laboratory Medicine, David Geffen School of Medicine, University of California, Los Angeles, Los Angeles, California, USA

^2^UCLA Clinical Genomics Center, David Geffen School of Medicine, University of California, Los Angeles, Los Angeles, California, USA

^3^Department of Physiology, Southern Illinois University School of Medicine, Carbondale, IL, USA

^4^Department of Cellular and Genetic Medicine, School of Basic Medical Sciences, Fudan University, Shanghai 200032, China

^5^Department of Human Genetics, David Geffen School of Medicine, University of California, Los Angeles, Los Angeles, California, USA

^6^Department of Pediatrics, David Geffen School of Medicine, University of California, Los Angeles, Los Angeles, California, USA

^7^ Kaiser Permanent Oakland, CA

^*^Correspondence to:

Samuel P. Strom, Ph.D, FACMG

Department of Pathology and laboratory Medicine

David Geffen School of Medicine

ADDRESS

University of California, Los Angeles

Los Angeles, USA

Phone: (310) 794 5632

Fax: (310) 825 3991

Email: [sstrom@mednet.ucla.edu](mailto:sstrom@mednet.ucla.edu)

**Supplementary Table 1.** *MEFV*, *DEAF1* and *MED13L* variants were identified through clinical exome sequencing

| **Variant** | | | **Position** | | **Variant Type** | | | | **Allele frequency*** | | | | **Putative impact**** | | **Conservation** | **Experimental evidence** | **Loss of function^+^** | | **ACMG guidelines** |
| --- | --- | --- | --- | --- | --- | --- | --- | --- | --- | --- | --- | --- | --- | --- | --- | --- | --- | --- | --- |
| Genomic  Change (hg19) | cDNA change | Protein  change | Location | Protein domain | *De novo* variant | Zygosity | Novel | | | ExAC | 1000 genomes | ESP5400 | Polyphen2 | SIFT | Conservation | Experimental evidence |  |  | |
| *MEFV*  Chr16:3299468 | c.1223G>A | p.Arg408Gln | Exon 3 | bBox zinc finger | Unknown | Het | | Yes | 0 | | 0 | 0 | Tolerated | Benign | + | + | - | Likely Pathogenic | |
| *MEFV*  Chr16:3299586 | c.1105C>T | p.Pro369Ser | Exon 3 | bBox zinc finger | Unknown | Het | | Yes | 0 | | 0 | 0 | Damaging | Possibly damaging | + | + | - | Pathogenic | |
| *MEFV*  Chr16:3304626 | c.442G>C | p.Glu148Gln | Exon 2 | None | Unknown | Het | | Yes | 0 | | 0 | 0 | Tolerated | Benign | + | + | - | Disease associated polymorphism | |
| *DEAF1*  Chr11:686974 | c.688C>G | p.Gln212Glu | Exon4 | SAND | Unknown | Het | | Yes | 0 | | 0 | 0 | Possibly damaging | Tolerated | + | - | + | PS3/PM1/PM2 | |
| *MED13L*  Chr12:116418637 | c.676C>T | p.Pro1761Leu | Exon 5 | None | Unknown | Het | | Yes | 0 | | 0 | 0 | Benign | Tolerated | + | - | + | PM3/PM6/PP2/PP3 | |
| *Minor allele frequencies listed are from ExAC browser (exac.broadinstitute.org), 1000 Genomes Project ([www.1000genomes.org](http://www.1000genomes.org)), ESP5400 data of the National Heart, Lung, and Blood Institute GO Exome Sequencing Project (<http://evs.gs.washington.edu/EVS>) **^+^ =**Loss of Function is predicted by Loss of Function Score from ExAC **Predicted by Polyphen2 (http://genetics.bwh.harvard.edu/pph2/index.shtml) and SIFT (http://sift.jcvi.org/), variant function interpreted according to the guidelines from the American College of Medical Genetics and Genomics (ACMG) [Richards et al., 2015] | | | | | | | | | | | | | | | | | | | |

| ****Supplementary Table** **2** Clinical Features of Patient Compared to *MED13L* cases in the literature** | | | |
| --- | --- | --- | --- |
|  | **Patient** | ***MED13L*  cases in the literature^1^** | |
| **Neurological and Developmental Abnormalities** |  |  | |
| **Intellectual disability** | **+** | **21/21** | |
| **Developmental delay** | **+** | **21/21** | |
| **Speech Development delay** | **+** | **10/13** | |
| **Hypotonia** | **+** | **13/13** | |
| **Infantile feeding difficulties** | **+** | **1/1** | |
| **Motor delay/Incoordination of Ataxia** | **+** | **15/15** | |
| **Seizures** | **+** | **1/16** | |
| **Behavioral Problems** |  |  | |
| **Autism/Autistic Behavioral Problems** | **+** | **9/9** | |
| **Pain Insensitivity** | **+** | **5/5** | |
| **Sleep Disturbances** | **+** | **1/1** | |
| **Cardiac Anomalies** | **+** | **8/8** | |
| **Craniofacial Abnormalities** |  |  | |
| **Craniofacial Manifestations** | **+** | **8/8** | |
| **Round Face** | **-** | **7/7** | |
| **Macrocephaly** | **-** | **2/16** | |
| **Brachycephaly** | **-** | **2/16** | |
| **Nasal Abnormalities** | **-** | **11/11** | |
| **Out Ear Abnormalities** | **+** | **4/4** | |
| **Nasal Bridge Abnormalities** | **-** | **3/3** | |
| **Open Mouth** | **+** | **13/13** | |
| **Prognathism** | **-** | **5/7** | |
| **Macroglossia** | **-** | **1/1** | |
| **1=**[Asadollahi et al., 2013; Utami et al., 2014; Asadollahi et al., 2014; Hamdan et al., 2014; Bulaveya et al., 2015; Codina-Sola et al, 2015; Redin et al., 2014; Adegbola et al., 2015] | | | |

**Supplementary Figure 1**

**
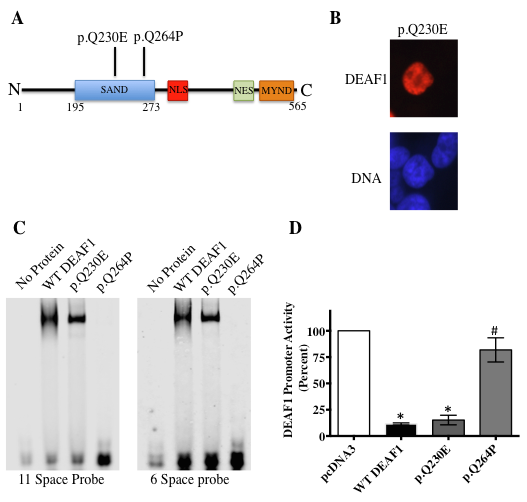
**

Supplementary Figure 1. Subcellular Localization Studies. Overexpression of p.Q230E in HEK293T cells showed predominantly nuclear subcellular localization similar to WT DEAF1.
